# The influence of self-generated song during aggression on brain serotonin levels in male crickets

**DOI:** 10.1093/jisesa/ieae097

**Published:** 2024-09-30

**Authors:** Masanori T Itoh

**Affiliations:** Department of Biology, Liberal Arts and Sciences Division, Institute of Education, Tokyo Medical and Dental University, Ichikawa, Chiba 272-0827, Japan

**Keywords:** aggressive song, brain, central body, cricket, serotonin

## Abstract

Pairs of adult male crickets, *Gryllus bimaculatus,* fight and immediately determine winner and loser statuses. The winner male repeatedly produces an aggressive (rival) song by rubbing his forewings together. In this study, I removed the plectrum, a sound-producing structure in the forewing, from male crickets and measured their brain serotonin (5-hydroxytryptamine: 5-HT) levels immediately after a 10-min aggressive interaction. Pairs of plectrum-removed males fought and established clear winner–loser relationships, like the case of intact males. The plectrum-removed winner males frequently rubbed their forewings together, but were unable to produce song. Aggressive interaction reduced significantly brain 5-HT levels in the plectrum-removed males, regardless of their winner and loser statuses. Furthermore, the reduction of brain 5-HT was detected primarily in the central body, a group of neuropils spanning the midline of the brain. In contrast, in pairs of intact males, aggressive interaction reduced brain 5-HT levels in the loser males, but not in the winner males. Plectrum removal alone did not affect the brain’s 5-HT levels. These results suggest that aggressive song emitted by the winner male cricket prevents the reduction of 5-HT levels in his own brain, especially in the central body.

## Introduction

Adult males of several insect species, including the cricket (*Gryllus bimaculatus*), generate several types of songs, such as aggressive (rival), courtship, and calling songs, by rubbing their wings together (Libersat et al. 1994, Pfau and Koch 1994). Wing songs have been thought to play important roles in intraspecific communication (Pfau and Koch 1994, Hoy et al. 1997, Doi et al. 2001, Poulet 2005). It has been demonstrated that males warn off rival males or attract females with wing songs (Pfau and Koch 1994, Hoy et al. 1997, Doi et al. 2001, Poulet 2005).

When 2 adult male crickets are placed together in a confined space, they tend to begin a series of encounter-specific behaviors, including grappling, mandible flaring, and biting (Adamo and Hoy 1994, Stevenson et al. 2000, Murakami and Itoh 2001, 2003). Immediately after fighting, a clear winner–loser relationship is formed, in which the winner male repeatedly displays aggressive behavior toward the loser male and emits an aggressive song by lifting his forewings and rubbing them together, while the loser male runs away from the aggressive opponent. The aggressive behavior displayed by the winner male is repeated as long as the 2 males are kept together, and the winner and loser statuses are maintained throughout the entire time (Adamo and Hoy 1994, Stevenson et al. 2000, Murakami and Itoh 2001, 2003).

We previously found that clear winner–loser relationships were established and maintained in pairs of male crickets whose forewings were entirely removed, similar to those seen for intact males, although the wingless winner male was unable to produce aggressive song (Murakami and Itoh 2001). This indicates the possibility that an aggressive song is not essential for establishing and maintaining winner–loser relationships. Moreover, aggressive interaction reduces the levels of the neurotransmitter serotonin (5-hydroxytryptamine: 5-HT) in the brains of the wingless males, regardless of their winner and loser statuses (Murakami and Itoh 2001). In contrast, in pairs of intact male crickets, aggressive interaction reduces brain 5-HT levels in the loser males, but not in the winner males (Murakami and Itoh 2001). Thus, aggressive song emitted by winner male crickets may affect the activity of 5-HT neurons in their own brains.

**Fig. 1.**
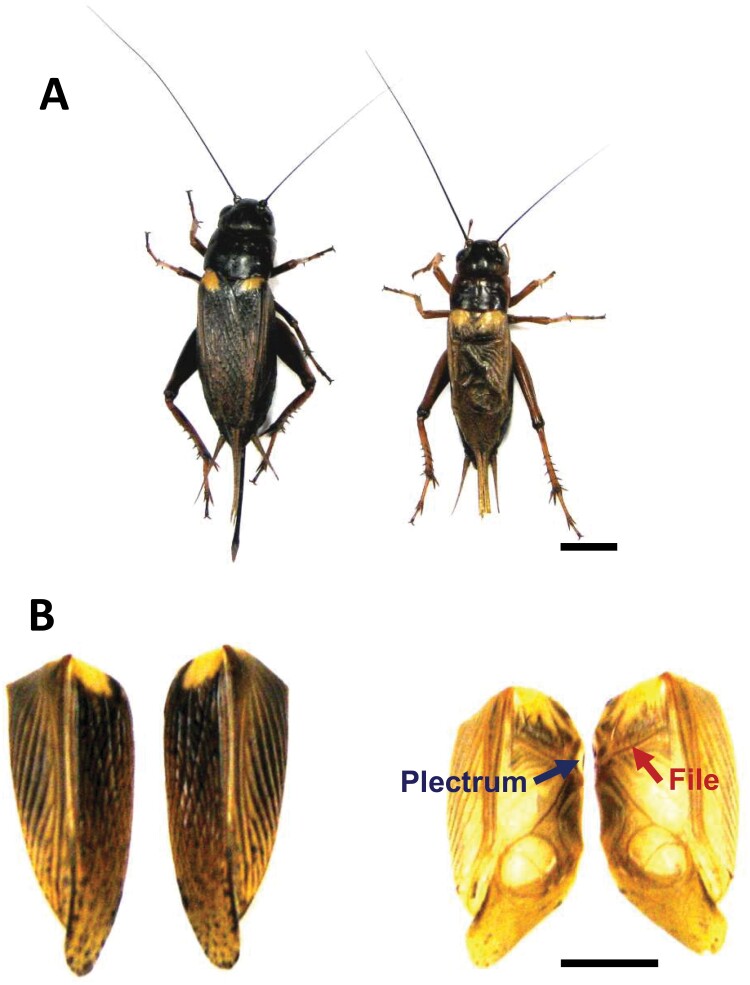
The forewings of adult crickets (*G. bimaculatus*). A) Intact male (right) and intact female (left). B) Dorsal surface of forewings of the male (right) and the female (left). The anterior is at the top. Blue and red arrows indicate plectrum on the left forewing and file on the right forewing, respectively. Sexual differences are visible in the forewing structures; e.g., plectrum and file are present only in the forewings of males. The male produces song by rubbing the forewings together so that the plectrum on a forewing is scraped along the ventral surface of the file on the other forewing. Scale bars in A) and B), 5 mm.

Even if the plectrum (a sound-producing structure in the forewing) of a male cricket (Fig. 1) is removed, his forewing movement (singing) behavior is unaffected (Poulet 2005). However, the movement no longer produces song; i.e., the male cricket performs “silent singing.” In this study, to assess whether aggressive song emitted by winner males affects their own brain 5-HT levels, I measured brain 5-HT levels of plectrum-removed and intact male crickets immediately after aggressive interaction.

## Materials and Methods

### Animals, Housing, and Treatment

Shortly after imaginal ecdysis, adult male crickets (*G. bimaculatus*) were taken from a colony that had been maintained in our laboratory. As previously described (Murakami and Itoh 2001, 2003), they were isolated for 7 days to minimize prior social experience by individually placing them in separate containers (6.0 cm diameter, 8.5 cm height). They did not see other crickets until the behavioral testing, but could potentially hear and smell conspecifics. They were maintained under controlled conditions of temperature (25 °C) and light (a 12 h light:12 h dark cycle; lights on from 06:00 to 18:00 h), with free access to food and water. After 7 days of isolation, some male crickets were anesthetized by placing them on ice for 7–10 min, and the plectrum (Fig. 1B) was then removed from the forewing using fine scissors. Thereafter, all crickets were maintained for 7 days under the above conditions, until the behavioral testing.

### Behavioral Testing and Brain Sampling

Adult male crickets used in behavioral tests were approximately equal in size [weighing 1.14–1.20 g (*n* = 50) in intact males and 1.10–1.18 g (*n* = 45) in plectrum-removed males]. As previously described (Murakami and Itoh 2001, 2003), 2 male crickets (*n* = 15 pairs/group) were simultaneously introduced into a glass container (10.0 cm diameter, 15.0 cm height), and their behaviors were monitored for a 10-min period. During the test period, the male who repeatedly chased and bit his opponent was judged as being winner, while the male who ran away from the aggressive opponent was judged as being loser. Each behavior is easily distinguished from others (Stevenson et al. 2000, Murakami and Itoh 2001, 2003). All behavioral tests were conducted at 25 °C during the late light time (15:00–18:00 h) of the light/dark cycle. After each trial, the glass container was washed, rinsed with ethanol, and air-dried. Immediately after each trial, the males were killed by decapitation. The brains were rapidly dissected out of the head capsule and were processed immediately or stored at −80 °C until use. As controls, individually housed males (intact males, *n* = 20; plectrum-removed males, *n* = 15) were placed into the glass container described above, without other crickets for 10 min, and the brains were removed immediately.

### High-Performance Liquid Chromatography (HPLC) for 5-HT

The brains (*n* = 5/group) were stored at −80 °C for no longer than 2 days before use. The 5-HT levels were determined by reverse-phase HPLC coupled with fluorometric detection, as previously described (Murakami and Itoh 2001, 2003). Each brain was homogenized by sonication in 100 μl of ice-cold 0.15 M perchloric acid containing 0.025% each of cysteine and disodium EDTA and was centrifuged at 20,000 × *g* for 10 min at 4 °C. The supernatant was passed through a 0.45-μm filter, and 25 μl of the filtrate was injected into an HPLC system equipped with a reverse-phase column (CAPCELL PAK C18 UG80; 250 mm × 4.6 mm internal diameter, 5-μm particles; Shiseido, Tokyo, Japan) and a fluorometric detector (RF-10A XL; Shimadzu, Kyoto, Japan). The fluorometric detector was used with the excitation and emission wavelengths set at 280 and 340 nm, respectively. The mobile phase consisted of 12.2 mM citric acid, 11.6 mM ammonium phosphate, 2.5 mM sodium octylsulfate, 3.3 mM dibutylamine phosphate, 1.1 mM disodium EDTA, and 7.5% acetonitrile (v/v) with pH adjusted to 3.8. Dibutylamine phosphate was prepared from dibutylamine and phosphoric acid (Lee Chin 1992). The mobile phase was run isocratically at a flow rate of 1.0 ml/min at 30 °C. Peaks on the HPLC chromatograms were identified from their retention times, and 5-HT was quantified according to its peak height. The authenticity of the 5-HT peak was verified by coelution with an authentic standard (Murakami and Itoh 2001). 5-HT and its structurally related compounds were obtained from Sigma-Aldrich (St. Louis, MO, USA), and prepared in 0.06 M perchloric acid containing 0.025% each of cysteine and disodium EDTA. Brain 5-HT levels are expressed as the mean ± SEM. Statistical comparisons of the group means of brain 5-HT levels were performed by independent and paired-sample *t*-tests. In all cases, *P* < 0.05 was considered statistically significant.

### 5-HT Immunohistochemistry

The experimental procedure for 5-HT immunohistochemistry was previously described (Murakami and Itoh 2003). Briefly, brains (*n* = 10/group) were rapidly dissected out in freshly prepared 2% paraformaldehyde in 0.1 M phosphate buffer (pH 7.4), and were fixed in this solution for 20 h at 4 °C. After routine tissue processing by dehydration through an ascending ethanol series, clearing in xylene, and infiltration with Paraplast Plus (Sigma-Aldrich), the brains were embedded in Paraplast Plus and serially sectioned at a thickness of 6 μm. The brain sections were mounted on slides, deparaffinized in xylene, and rehydrated through a graded series of ethanol to distilled water. After washing with 10 mM phosphate-buffered saline containing 0.1% Tween 20 (PBS/Tween, pH 7.4), endogenous peroxidase activity was inactivated by incubation in 0.03% hydrogen peroxide (Dako, Carpinteria, CA, USA) in methanol for 20 min at 24 °C. Nonspecific background in the tissue sections was blocked by incubation with 1.0% bovine serum albumin in PBS/Tween for 1 h at 24 °C and was then incubated for 18 h at 4 °C with a polyclonal rabbit antibody against 5-HT (Chemicon International Inc., Temecular, CA, USA) at a dilution of 1:1000 in PBS/Tween. The specificity of this antibody has been documented (Steinbusch et al. 1978). The sections were washed with PBS/Tween, the sections were incubated with peroxidase-labeled polymer conjugated to goat anti-rabbit immunoglobulin (Dako) for 30 min at 24 °C. Subsequently, the immunoreactivities were detected using 3,3´-diaminobenzidine (Dako) as a substrate. The chromogen detection reaction was performed for 20 s at 24 °C and was stopped by washing in distilled water. The sections were counterstained with hematoxylin (Dako), dehydrated, cleared, and mounted in Entellan (Merck KGaA, Darmstadt, Germany) under glass coverslips. Omission of the antibody against 5-HT or preincubation of the 5-HT antibody with 5-HT prevented immunolabeling.

## Results

**Fig. 2.**
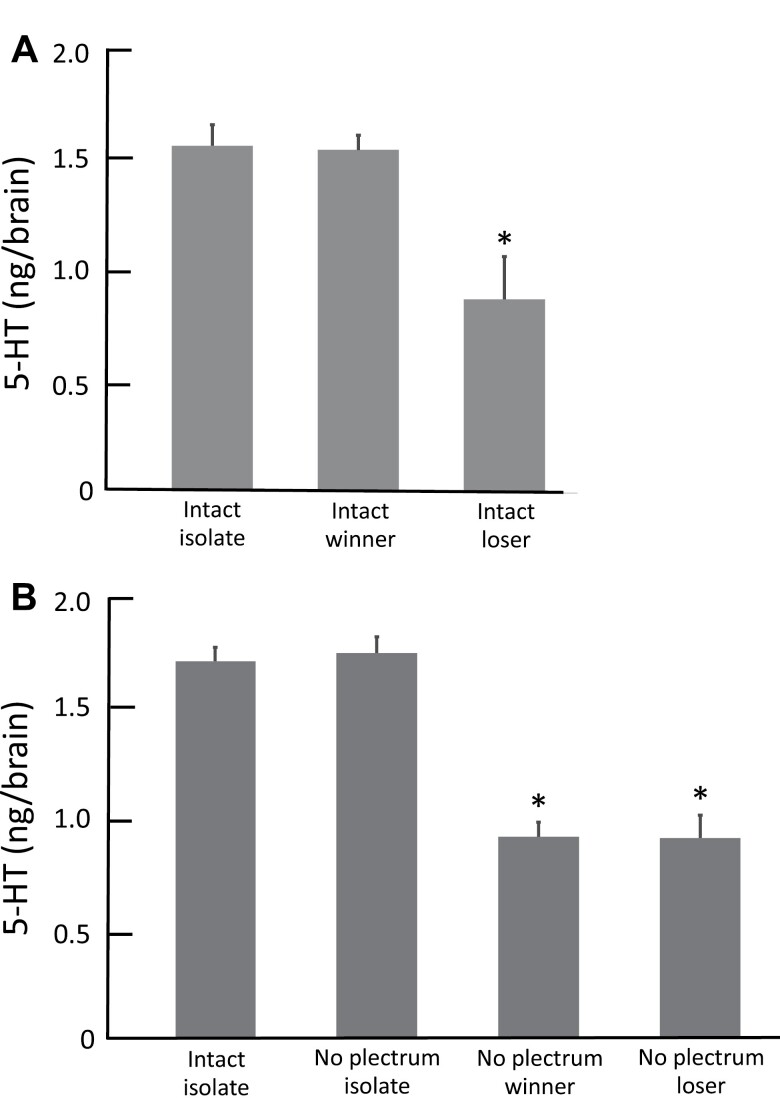
A) Effect of aggressive interaction on brain 5-HT levels in intact male crickets (*G. bimaculatus*). Intact isolate, intact males isolated individually; intact winner, intact winner males; and intact loser, intact loser males. The brains in intact winner and intact loser groups were rapidly removed immediately after a 10-min aggressive interaction. B) Effects of plectrum removal and aggressive interaction on brain 5-HT levels in male crickets (*G. bimaculatus*). Intact isolate, intact males isolated individually; no plectrum isolate, males who had been removed the plectrum (a sound-producing structure in the forewing) and individually isolated; no plectrum winner, plectrum-removed males who were winner; and no plectrum loser, plectrum-removed males who were loser. The brains in no plectrum winner and no plectrum loser groups were rapidly removed immediately after a 10-min aggressive interaction. In A) and B), the 5-HT levels in each brain were determined by HPLC with fluorometric detection. For other experimental conditions, see *Materials and Methods* section. Data represent means ± SEM (*n* = 5 crickets per group). In A), * *P* < 0.001 vs. levels in intact isolate and intact winner groups. In B), *P < 0.001 vs. levels in intact isolate and no plectrum isolate groups.

There was no significant difference in brain 5-HT levels between the intact winner males and the intact isolated males (*n* = 5; *t *= 0.232, *P* = 0.411; Fig. 2A). Immediately after a 10-min aggressive interaction, brain 5-HT levels of intact loser males were significantly lower than those of intact winner males (*n* = 5; *t* = 5.787, *P* < 0.001; Fig. 2A).

When plectrum-removed males and intact males were individually housed, there was no significant difference in brain 5-HT levels between these 2 groups (*n* = 5; *t* = 0.785, *P* = 0.228; Fig. 2B), indicating the possibility that plectrum removal alone did not affect the brain 5-HT levels. When pairs of plectrum-removed males were placed together, fighting was observed in all pairs (*n* = 15), similar to that observed in the pairs of intact males. Immediately after the fight, a clear winner–loser relationship was established. The plectrum-removed, winner males repeatedly chased and bit the loser ones during the 10-min test period, indicating that plectrum-removed pairs maintained their winner–loser relationships during the entire test period. In addition, the plectrum-removed dominant males frequently lifted their forewings and rubbed them together, just like the intact winner males. However, the plectrum-removed winner males were unable to produce sound.

Immediately after the 10-min aggressive interaction, the brain 5-HT levels in plectrum-removed winner males were significantly lower than those of plectrum-removed and individually isolated males (*n* = 5; *t* = 16.914, *P* < 0.001; Fig. 2B), as well as in plectrum-removed loser males (*n* = 5; *t* = 13.172, *P* < 0.001; Fig. 2B). There was no significant difference in brain 5-HT levels between the plectrum-removed winners and the plectrum-removed losers (*n* = 5; *t* = 0.098, *P* = 0.462; Fig. 2B), unlike that observed in intact males (Fig. 2A).

**Fig. 3.**
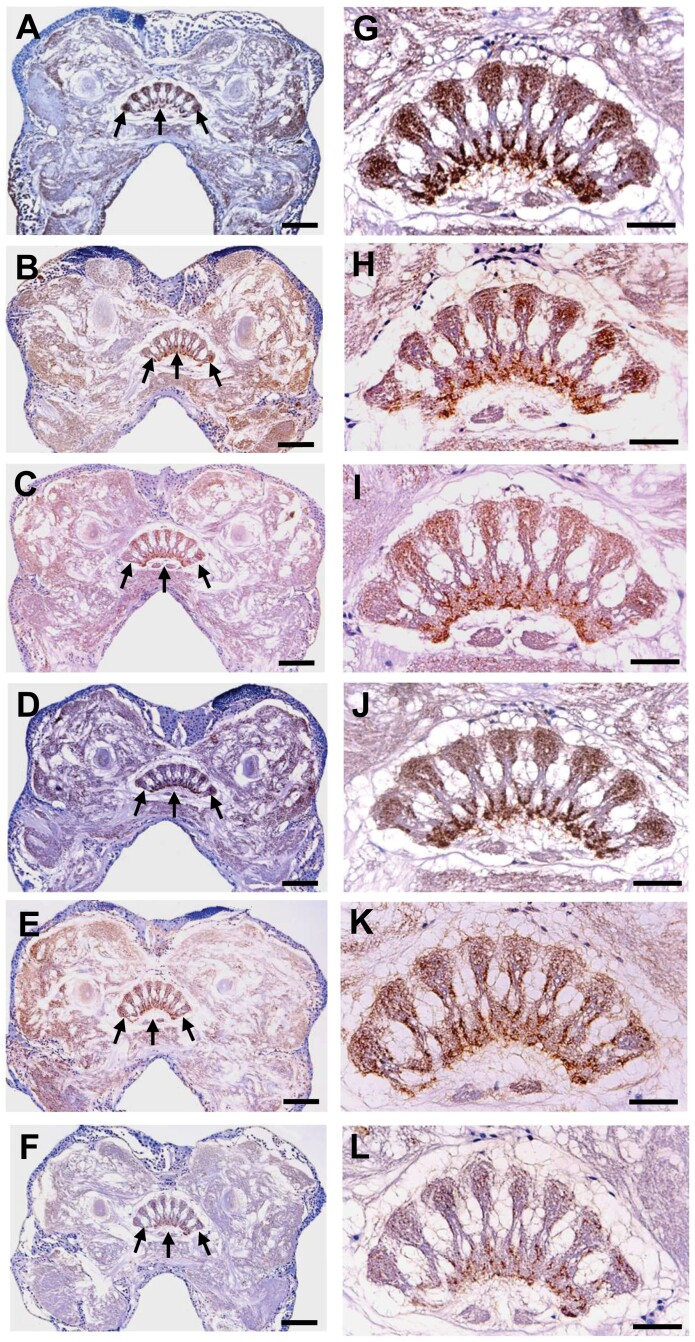
5-HT immunoreactivity in frontal sections of the male cricket (*G. bimaculatus*) brains. Ten brains in each group were subjected to 5-HT immunohistochemistry. The brains of winner and loser males were rapidly removed immediately after a 10-min behavioral test. 5-HT immunoreactivity is indicated by brown coloration. The sections were counterstained with hematoxylin, which is indicated by dark blue coloration. For other experimental conditions, see *Materials and Methods* section. Micrographs indicate the resulting representative sections of each group. A) and G), intact male isolated individually; B) and H), intact winner male; C) and I), intact loser male; D) and J), plectrum removed male isolated individually; E) and K), winner male with the plectrum removed; and F) and L), loser male with the plectrum removed. A)–F) show almost the same plane of brain section, and the arrows show the central body. G)–L) show higher magnification of the central body in A)–F), respectively. Dorsal is to the top. Scale bars in A)–F), 150 μm; in G)–L), 50 μm. Scale bars in A)–F), 150 μm; in G)–L), 50 μm. In intact isolated males and intact winner males, strong 5-HT immunoreactivity was observed predominantly in the central body (A, B, G, and H). In contrast, 5-HT immunoreactivity was markedly reduced in the central body of intact loser males (C and I). Plectrum removal alone did not affect 5-HT immunoreactivity in the central body (D and J). The reduction of 5-HT immunoreactivity was observed in the central body of plectrum-removed males regardless of their winner and loser statuses (E, F, K, and L).

Immunohistochemical analysis revealed that 5-HT-immunoreactive neurons were distributed widely throughout the brains of male crickets (Fig. 3). In intact isolated males and intact winner males immediately after the behavioral test, strong 5-HT immunoreactivity was observed predominantly in the central body, a group of neuropils spanning the midline of the brain (Fig. 3A, B, G, and H). In contrast, 5-HT immunoreactivity was markedly reduced in the central body of intact loser males (Fig. 3C and I). Plectrum removal alone did not affect 5-HT immunoreactivity in the central body (Fig. 3D and J). The reduction of 5-HT immunoreactivity was observed in the central body of plectrum-removed males regardless of their winner and loser statuses (Fig. 3E, F, K, and L).

## Discussion

In the present study, pairs of plectrum-removed males fought and established clear winner–loser relationships, like the case of intact males. The plectrum-removed pairs maintained their winner–loser relationships during the entire test period. The plectrum-removed winner males repeatedly rubbed their forewings together, but they were unable to produce song. On the basis of these results, I indicate the possibility that aggressive song emitted by winner male crickets is not required for the formation and maintenance of winner–loser relationships, which is consistent with previous studies using male crickets whose entire forewings were removed (Murakami and Itoh 2001).

In the current study, aggressive interaction reduced significantly brain 5-HT levels in plectrum-removed males, regardless of their winner and loser statuses. The reduction of 5-HT was detected preliminarily in the central body of the brain. In contrast, in pairs of intact males, aggressive interaction reduced significantly brain 5-HT levels in the loser males, but not in the winner males. In addition, plectrum removal alone did not affect brain 5-HT levels of male crickets. These results suggest that aggressive song emitted by the winner male cricket prevents the reduction of 5-HT levels in his own brain, especially in the central body. Wing songs, including aggressive song, have been thought to play important roles in intraspecific communication (Pfau and Koch 1994, Hoy et al. 1997, Doi et al. 2001, Poulet 2005). In addition, the present results suggest that in male crickets, self-generated song affects brain functions involving synthesis and/or degradation of 5-HT in neurons of the central body, resulting in alteration of the synaptic transmission.

It has been suggested that the central body is involved in memory formation of courtship conditioning (Joiner and Griffith 2000) and wing-guided olfactory navigation (Matheson et al. 2022) in the fruitfly *Drosophila*, singing behavior in the grasshopper *Chorthippus biguttulus* (Heinrich et al. 2001, Wenzel et al. 2005), and sky compass orientation in the desert locust *Schistocerca gregaria* (Vitzthum et al. 2002, Heinze et al. 2009) and the monarch butterfly *Danaus plexippus* (Heinze and Reppert 2011).

5-HT neurons of the central body in *Drosophila* mediate the effects of 5-HT on sleep fragmentation (Liu et al. 2019). However, the physiological role of 5-HT neurons in the cricket central body is unknown. We previously found that 7 days after the removal of antennae, 5-HT levels in the central body of male crickets were reduced, and that the male crickets with antennae removed failed to display male-male aggression and displayed courtship behavior toward other males (Murakami and Itoh 2001).

Brain 5-HT has been demonstrated to be associated with both aggression and dominance in invertebrates (Dyakonova et al. 1999, Stevenson et al. 2000, Murakami and Itoh 2001, Kravitz and Huber 2003, Dierick and Greenspan 2007, Dyakonova and Krushinsky 2013) as well as vertebrates (Blanchard et al. 1991, Summers et al. 1998, van der Vegt et al. 2003, Nelson et al. 2006). The present and previous studies indicated that aggressive interaction reduced brain 5-HT levels in intact loser male crickets, but not in intact winner male crickets (Murakami and Itoh 2001). During and immediately after aggressive interaction, loser male crickets are unaggressive relative to dominant males (Murakami and Itoh 2001, Hofmann and Stevenson 2010). In addition, treatment of male crickets with the 5-HT synthesis inhibitor α-methyltryptophan reduces the likelihood that they will become dominant and increases the likelihood that they will exhibit loser-like escape responses (Dyakonova et al. 1999, Stevenson et al. 2000). Thus, reduced brain 5-HT levels in male crickets appear to be associated with decreased aggressiveness. In the present study, I found that aggressive interaction reduced significantly brain 5-HT levels in the plectrum-removed winner males, unlike those seen in intact winner males. Nevertheless, an obvious decrease in aggressiveness of the plectrum-removed winners was not seen as compared to that of the intact winners. Thus, it appears that brain 5-HT is not crucial for the regulation of aggressiveness in male crickets, although further careful observation of their behavior is needed. It has been suggested that octopamine (the invertebrate analog of noradrenaline) and dopamine are involved in aggressiveness in male crickets (Adamo and Hoy 1994, Stevenson and Schildberger 2013, Rillich and Stevenson 2014).

In summary, the present study suggests that aggressive song emitted by the winner male cricket prevents the reduction of 5-HT levels in his own brain, especially in the central body. It is also suggested that in aggressive male crickets, self-generated song affects brain functions such as synthesis and/or degradation of 5-HT in neurons of the central body, resulting in alteration of synaptic transmission. Further studies are necessary to examine the functional role of 5-HT neurons in the central body. The mechanism by which aggressive song emitted by dominant male crickets prevents the reduction of 5-HT levels in their own central body also needs to be clarified.
